# [Corrigendum] METTL3‑mediated m6A modification of Bcl‑2 mRNA promotes non‑small cell lung cancer progression

**DOI:** 10.3892/or.2023.8502

**Published:** 2023-02-15

**Authors:** Yongxi Zhang, Shuyuan Liu, Tiesuo Zhao, Chengxue Dang

Oncol Rep 46: 163, 2021; DOI: 10.3892/or.2021.8114

Subsequently to the publication of the above paper, the authors have realized, upon reorganizing all their original data, that errors were made during the assembly of the images in Fig. 5 on p. 8. Specifically, in Fig. 5E, the images intended to represent the ‘METTL3 sh-METTL3’ and ‘Bcl-2 sg-METTL3’ immunohistochemistry staining experiments were selected incorrectly.

The revised version of Fig. 5, showing the correctly assembled data panels for the ‘METTL3 sh-METTL3’ and ‘Bcl-2 sg-METTL3’ experiments in Fig. 5E, is shown on the next page. The authors sincerely apologize for the errors that were inadvertently introduced during the preparation of this Figure, thank the Editor of *Oncology Reports* for allowing them the opportunity to publish this Corrigendum, and regret any inconvenience that these errors may have caused to the readership.

## Figures and Tables

**Figure 5.**
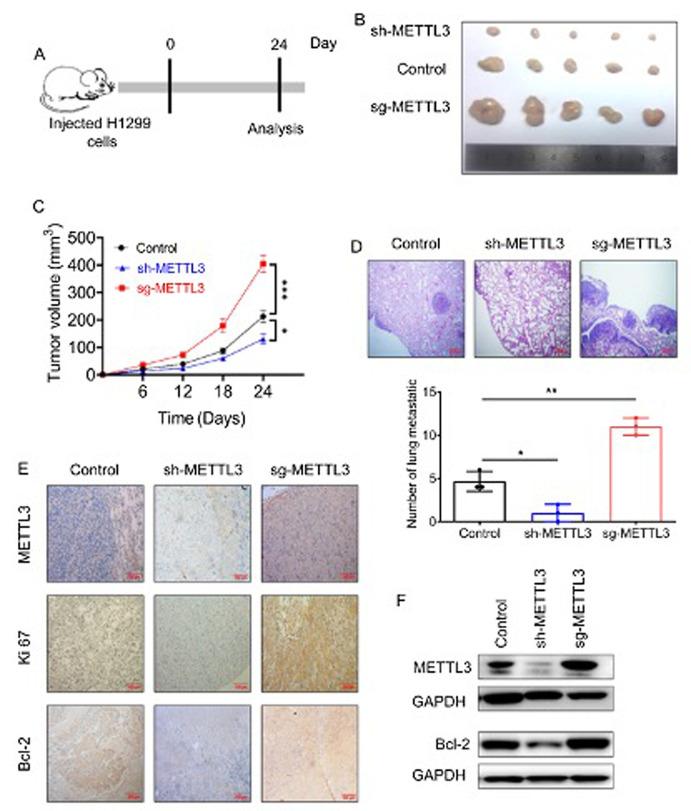
METTL3 promotes non-small cell lung cancer (NSCLC) cells proliferation *in vivo.* (A) A flow chart of experimental design *in vivo*. (B) An *in vivo* xenograft assay was performed using the H1299 cells transfected with METTL3 stable silencing (sh-METTL3), METTL3 stable overexpression (sg-METTL3) or control. (C) Quantitative analysis of xenografted tumor volume. (D) Histopathological examination of the lung tissue sections (n=5). (E) The expression of METTL3, Ki67 and Bcl-2 was detected by IHC assays in paraffin-embedded tissue (n=5). (F) The protein expression levels of METTL3 and Bcl-2 were examined via western blotting in implanted tumors (n=5). Error bars, SD. **, P<0.01; and ***, P<0.001.

